# Artificial intelligence and machine learning in cancer imaging

**DOI:** 10.1038/s43856-022-00199-0

**Published:** 2022-10-27

**Authors:** Dow-Mu Koh, Nickolas Papanikolaou, Ulrich Bick, Rowland Illing, Charles E. Kahn, Jayshree Kalpathi-Cramer, Celso Matos, Luis Martí-Bonmatí, Anne Miles, Seong Ki Mun, Sandy Napel, Andrea Rockall, Evis Sala, Nicola Strickland, Fred Prior

**Affiliations:** 1 Department of Radiology, Royal Marsden Hospital, Sutton, UK; 2 Champalimaud Foundation, Lisbon, Portugal; 3 Charité – Universitätsmedizin Berlin, Berlin, Germany; 4 Department of Surgery & Interventional Science, University College London, London, UK; 5 Department of Radiology and Institute for Biomedical Informatics, University of Pennsylvania, Philadelphia, USA; 6 Centre for machine learning, Massachusetts General Hospital/Harvard Medical School, Boston, USA; 7 Department of Radiology, Hospital Universitari i Politècnic La Fe, Valencia, Spain; 8 Department of Psychological Sciences, Birkbeck University, London, UK; 9 Arlington Innovation Center for Health Research, Virginia Tech, Arlington, USA; 10 Department of Radiology, Stanford University, Stanford, USA; 11 Department of Radiology, Imperial College Healthcare NHS Trust, London, UK; 12 Department of Radiology, Cambridge University, Cambridge, UK; 13 Department of Biomedical Informatics and Department of Radiology, University of Arkansas for Medical Sciences, Little Rock, USA

**Keywords:** Cancer imaging, Biomarkers

## Abstract

An increasing array of tools is being developed using artificial intelligence (AI) and machine learning (ML) for cancer imaging. The development of an optimal tool requires multidisciplinary engagement to ensure that the appropriate use case is met, as well as to undertake robust development and testing prior to its adoption into healthcare systems. This multidisciplinary review highlights key developments in the field. We discuss the challenges and opportunities of AI and ML in cancer imaging; considerations for the development of algorithms into tools that can be widely used and disseminated; and the development of the ecosystem needed to promote growth of AI and ML in cancer imaging.

## Abstract (web summary)

Koh, Papanikolaou et al. discuss the application of artificial intelligence in cancer imaging. The authors highlight opportunities for exploiting machine learning algorithms in this field, and outline barriers in their implementation and how these might be addressed.

## Introduction

Artificial intelligence (AI) and machine learning (ML) are rapidly transforming the scientific landscape, including many domains in medicine. AI refers to the creation of machines or tools that can simulate human thinking and behaviour, whereas ML is a subset of AI in which machine or tools learn from data to make classifications or prediction either with or without human supervision^1^. The advancement in these fields in recent years has been accelerated by the emergence of high performance computers.

In medicine, digitised domains, such as imaging, lend themselves to become early adopters of AI and ML. The imaging pipeline from image acquisition, reconstruction, interpretation, reporting and the communication of results is operated within the digital space, allowing such data to be effectively captured for AI and ML. In particular, as cancer imaging represents a substantial proportion of the work in many departments, it is an area where early exploration and adoption of these technologies by radiologists as primary users appear likely. This is especially the case since these tasks can be repetitive (such as in cancer screening, where readers need to sieve through a large volume of normal studies to identify abnormalities), tedious (such as serial tumour measurements) and burdensome (such as outlining tumours for disease segmentation). Indeed, there are already a number of extant commercial products in the cancer imaging space, with the aim of improving work efficiency, reducing errors, and enhancing diagnostic performance.

Many technological solutions are being developed in isolation, however, which may struggle to achieve routine clinical use. These may have been hampered by the limited opportunities for clinicians, radiologists, scientists, and other experts to interact collectively to understand the clinical and data science landscape; to identify the needs, risks, opportunities and challenges for the development, testing, validation and adoption of such tools. This requires the nurturing of multidisciplinary ecosystems collectively, including commercial partners as appropriate, to drive innovations and developments.

This review aims to foster interdisciplinary communication on the above issues. We outline relevant AI and ML techniques and highlight key opportunities for implementing AI and ML in cancer imaging. The clinical, professional and technical challenges of implementing AI and ML in cancer imaging are discussed. We draw upon lessons learnt from the past, and take a forward look into the technical and infrastructural developments that are needed to facilitate AI in cancer imaging, enabling the integration of AI and ML technologies into hospital systems and the appropriate training of the future workforce.

### The medical image as imaging data: Radiomics

Medical images are still largely evaluated by expert radiologists, who are able to visually assess the absence or presence of disease, delineate the boundaries of tumours, evaluate tumour response to treatment and identify disease relapse. These human skills are generally used to define the reference standards against which AI and ML techniques are evaluated. However, there is increasing interest in exploring the smaller subunits that make up medical images (pixels/voxels) as imaging data, which lend themselves to analysis by computers to discover objective mathematical features that may be linked to disease behaviour or outcomes.

**Fig. 1:**
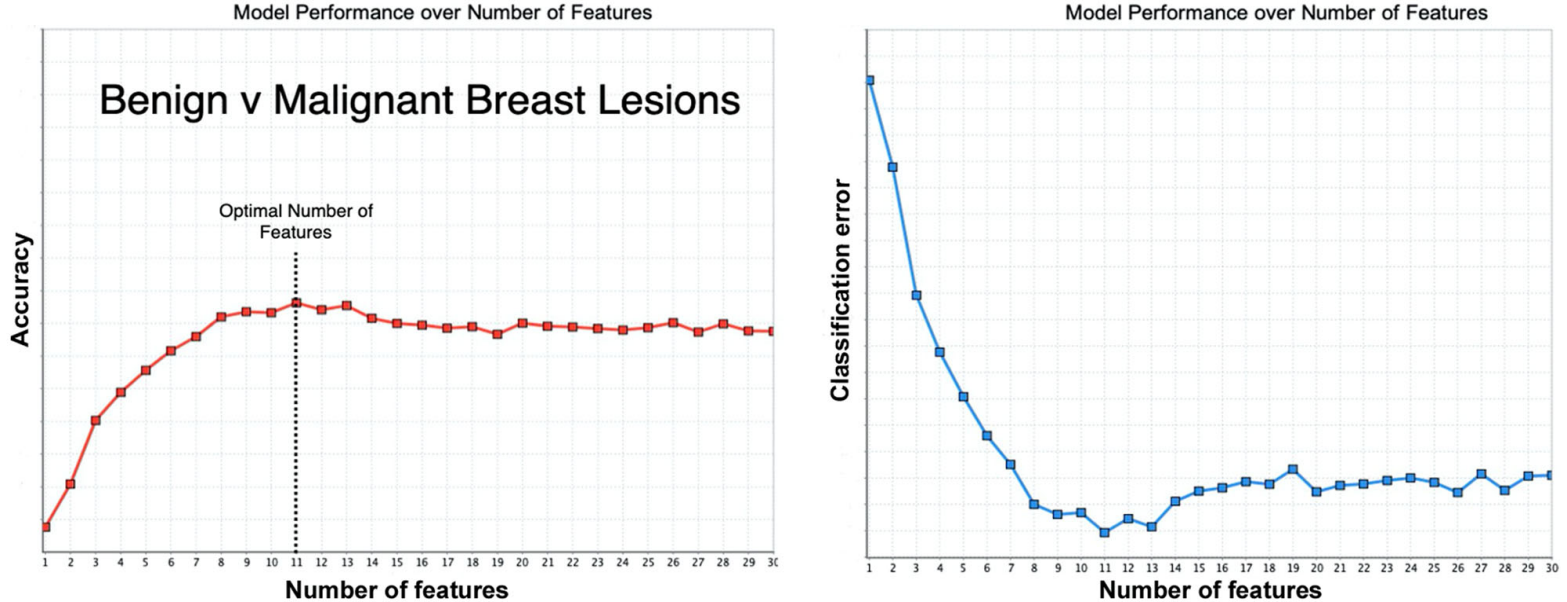
Feature selection for radiomics. In this illustration, a model classifier is shown to differentiate benign from malignant breast lesions on imaging. Initially, a large number of radiomic features were computed and after removing the highly correlated features, the zero and near-zero variance features; a recursive feature elimination and reduction method was applied. The model performance illustrated here identifies11 features to be at the saturation point. The red curve (left) is showing accuracy versus number of features, while the blue curve (right) represents the model’s error function over the number of features. In this example, using 11 imaging features shows high accuracy while minimising the error function.

Radiomics is the computerized analysis of medical images, or regions within medical images^2^. The images can be multidimensional, e.g., 2D X-ray, 3D computed tomography (CT), 4D ultrasound; and scalar-, e.g., CT, where the CT value is directly related to the tissue electron density, or vector-valued, e.g. phase-contrast magnetic resonance imaging (MRI), where the measured MRI signal is related to a mathematical vector function. The main goal in radiomics is to utilize algorithms that can identify patterns within images—usually beyond those that the human eye can perceive—and to exploit them to make predictions and therefore aid the clinical decision-making process. The computerized processing of images usually leads to a large number of imaging features. However, it is the non-redundant, stable and relevant features that are selected to develop a mathematical model that will answer the relevant clinical question, the so-called ground truth variable. Figure 1 illustrates the selection and testing of radiomics features to determine their ability, in a specific use-case, to distinguish between benign and malignant breast lesions. As a further extension, radiogenomics approaches, which integrate both radiomics and genomics analyses, are being developed to provide integrated diagnostics to aid disease management^3,4^.

Another example of a data set for radiomics analysis is a volumetric chest CT scan containing a tumour (e.g. a lung nodule), and a typical workflow could include: (1) identification of the tumour within the scan; (2) annotation of the tumour with semantic features (usually by expert radiologists)^5^; (3) outlining or segmentation of the tumour^6^; (4) computation of pre-determined tumour features (e.g. size, mean intensity, image texture, shape, margin sharpness)^7–9^ and/or using automated learning for task-relevant features; and (5) building a classifier that uses the computed features to predict a clinical state, e.g., probability of a specific gene mutation, response to therapy or overall survival^10,11^.

**Fig. 2:**
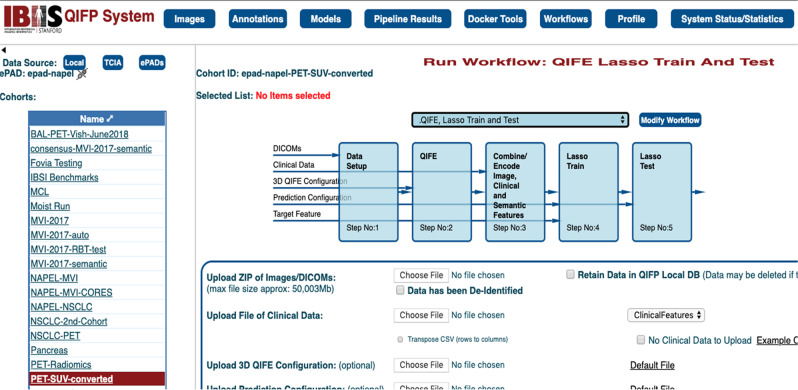
Quantitative Imaging Feature Pipeline. This shows an example of the quantitative imaging feature pipeline (QIFP) used to process a positron emission tomography (PET) imaging cohort stored on a local network ePAD server. The box next to the “modify workflow” button is a selection button, which has been set to choose the workflow displayed. This workflow moves the image data into Stanford’s Quantitative Image Feature Engine (QIFE)^64^, which computes thousands of image features for each segmented tumour in the cohort, followed by a sparse regression modeler (LASSO TRAIN) that derives an association between a linear combination of a small number of image features to 5-year survival, and finally tests that model in an unseen cohort and produces an ROC curve displaying the accuracy of the association. Other workflows can be chosen that use one or more of the existing tools stored on the QIFP system.

Several groups are building radiomics processing tools to facilitate pipeline data analysis. At Stanford, the Quantitative Image Feature Pipeline^12^ has been developed, which contains an expandable library of quantitative imaging feature extraction and predictive modelling algorithms, capable of comprehensive characterization of the imaging phenotype, and cloud-based software for creating and executing quantitative image feature-generating and predictive pipelines, and for using and comparing image features to predict clinical and molecular features. It also allows users to upload their own algorithms as Docker containers^13^, and to configure them in a customizable workflow (Fig. 2).

### AI and ML techniques in cancer imaging

In cancer imaging, images acquired from patients are pre-processed and transformed (to ensure data conformity or uniformity) as inputs to develop ML algorithms and models. Such pre-processing steps are used whether they relate to radiologist-defined features or mathematically derived radiomics features. This involves ensuring that the images are of similar image section thickness and of similar pixel-dimensions. As an overview, an ML model or algorithm maps the input imaging data and learns a simple or complex mathematic function that is linked to the target or output, such as a clinical or scientific observation. An ML algorithm can be established or trained with or without the use of so-called ground truth variables, which are reference findings verified by domain experts or by other means (e.g. pathology, laboratory tests, clinical follow-up). ML algorithms are usually developed using a training dataset, refined using a validation dataset, and then tested for their performance in an independent test dataset, ideally from a different institution.

Some types of ML models are more widely used than others in imaging studies. As a simplistic discussion, (assuming that *x* is the input variable, *f* the mathematic function and *y* the target/output variable), the most common form is the predictive model, where one tries to predict y by learning the *f(x)*. In exploratory models, one may simply attempt to link the input data *x* (e.g. an imaging feature) with the output *y* (e.g. gene expression).

When working with continuous variables, regression models, such as Linear, Cox (Proportional Hazards), Regression Trees, Lasso, Ridge, ElasticNet, or others can be used^14,15^. As for discrete variables, classification models such as Naïve Bays, Support Vector Machines, Decision Trees, Random Forests, KNN (k-nearest neighbours), Generalized Linear Models, Bagging and others can be used^16^. These models can inform cancer diagnosis, disease characterization and stratification, treatment response or disease outcomes^17^.

The success of any ML algorithm is influenced by data availability, machine computational power and subsequent algorithm refinements. The choice of ML algorithm may depend on data size. With smaller datasets (e.g. <1000 patients/examinations/images depending on use case), classical ML algorithms, such as Naïve Bayes, logistic regression, decision trees and support vector machines, are often applied. With larger datasets, more complex ML models, such as convolutional neural networks (CNN) that are very efficient in learning directly from images, may be preferable, although such models are more demanding in terms of computational power. CNN represent a type of deep learning, a subset of ML methods based on artificial neural networks. Artificial neural networks are inspired by the organization of neurons in the brain, simulating the connectivity of neurons to solve problems. ML algorithms can be supervised (i.e. the algorithm is developed using data that are labelled with some type of ‘correct answer’) at one end of the spectrum, or unsupervised (i.e. the algorithm uses the data to discover information by itself) at the other. The latter are associated with more complex CNN algorithms, which are able to discover patterns within imaging data without human intervention.

The driving force of CNNs has emerged from the computer vision domain, where the large dataset of ImageNet^18^ (a library of labelled photographic images) and the interest by internet developers to identify objects automatically on photographic pictures led to the development of very efficient ML architectures (e.g. Inception V3, AlexNet, VGG-16 and 19); Some of these have shown value for medical applications using a method called transfer learning^3^, where a pretrained architecture trained using ImageNet is then applied to medical imaging and fine-tuned for the specific use case.

In ML-based cancer imaging, it is not unusual for the number of predictors (e.g. CNN-derived features) to outweigh the number of data points or samples (patients or imaging studies). The latter results in model overfitting, where the model is optimized for the training dataset but consequently performs poorly on the test dataset. The most common strategies to reduce or prevent overfitting include: (a) to use techniques such as k-fold cross-validation by using multiple sub-samples of the dataset; (b) to train the algorithm with more data, where possible; (c) to perform feature selection, as appropriate, to reduce the dimensionality/number of the initial features; and/or (d) to implement ensemble learning, where feasible, to increase data size, that is to undertake algorithm training at multiple sites/institutions. Although an increasing number of healthcare organizations are moving to the cloud and centralized facilities to host and exploit their data, there is still resistance to data sharing and the need to protect patient privacy. These issues have fuelled distributed or federated learning approaches^19,20^. In federated learning, instead of collecting all data to a centralized repository, the models are circulated to different institutions and the models trained using local data at each site, sharing only the so called weights of a model between institutions. There is now also significant interest in the explainability^21^ and interpretability of algorithms to increase their trustworthiness. Clinical users may be less interested in the inner mechanics of ML models but would like to understand the way a model generates its output or prediction at a patient cohort level, as well as at an individual patient level.

#### Clinical opportunities for AI/ML in cancer imaging

**Fig. 3:**
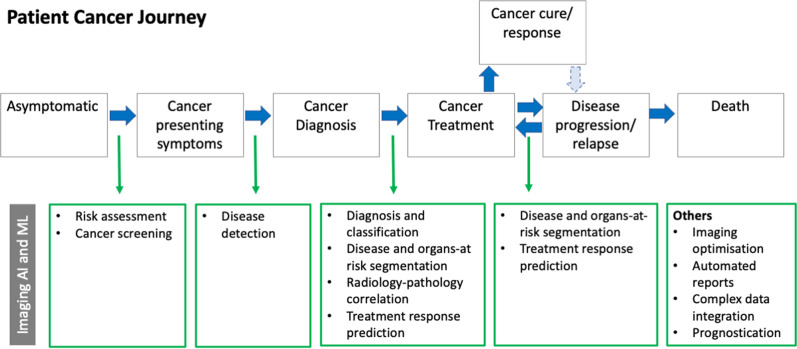
Potential use cases for artificial intelligence (AI) and machine learning (ML) in cancer imaging in relation to a patient’s cancer journey. A typical asymptomatic patient eventually develops cancer presenting symptoms, which usually leads to the cancer diagnosis. Following appropriate disease staging, cancer treatment commences, which can lead to good response or even cure. However, some patients will relapse or progress on treatment for which additional treatment may be administered. Unfortunately, some patients will succumb to their disease. The potential uses for Imaging AI and ML are as shown at various stages of the cancer journey and discussed in the text.

Machine learning can be harnessed in multiple ways to advance and improve cancer imaging. Figure 3 illustrates the typical clinical journey of a patient with cancer and highlights some of the key aspects of imaging where AI systems could exert a positive impact^22^. Here, we outline some of these in more detail.

##### Risk assessment

The optimal use of cancer imaging technologies requires that we direct resources to patients at greatest risk. In the US, many states require assessment of breast density to assess risk for developing cancer. A deep learning system has shown high accuracy in classifying breast density, and such systems will help support consistent density notification to patients in breast cancer screening^23,24^. This is particularly valuable since visual breast density measurement has been shown to be associated with considerable interobserver variations (6–85%)^25^. The use of AI-based approaches can improve upon current risk models. For example, a deep learning model that incorporated mammographic features and traditional risk factors to determine those at greatest risk for malignancy performed more effectively than conventional breast cancer risk models alone^26,27^. More recently, very good agreement was reported for breast cancer risk evaluation using mammographic breast density determined by a senior radiologist, a junior radiologist and an AI software^28^.

##### Cancer screening and cancer detection

Cancer screening has been a highly active area of AI research. AI algorithms have been tested in diseases with active screening programmes such as lung cancer^29–31^ and breast cancer^32–36^. In breast cancer, some studies have shown that AI algorithms can equal the performance of expert readers^36^, be used as a second reader for screening mammographic reviews^33^, provide triaging for prioritizing image reading^34^ and have been found to be acceptable to women undergoing mammographic screening^37^. However, real-world evidence is still insufficient to recommend the wide adoption of AI-based tools for breast screening^38^. In addition to systematic screening, opportunistic screening (the detection of abnormalities in exams obtained for other purposes) may create possibilities to detect other cancers, especially where directed screening tests would be impractical or cost ineffective. For example, in patients undergoing low-dose CT for lung cancer screening, it is possible to use the same images to assess breast cancer risk by assessing the breast density on CT^39^.

AI systems are now available for the detection of pulmonary nodues^31^, which also includes nodule classification, nodule measurement and malignancy prediction. When radiologists used a deep learning model for detection and management of pulmonary nodules, their performance improved and reading time was reduced^40^. Undoubtedly, the use case for AI in cancer detection will widen to include other tumour types.

##### Diagnosis and classification

ML systems provide ways to improve classification of imaging findings related to cancer. Malignant brain tumours have different aetiologies and prognosis, but tissue sampling is invasive and may not provide accurate characterization due to disease heterogeneity. Studies have shown the potential of AI to identify and classify major intracranial tumours, which include variously high grade gliomas, low grade gliomas, cerebral metastases, meningiomas, pituitary adenoma and acoustic neuromas, as well as differentiating these from normal tissues^41–44^. Another developing application in this area is the classification of cystic lesions of the pancreas, since distinguishing between intraductal papillary mucinous neoplasms, mucinous cystic neoplasm and serous cystic neoplasms of the pancreas can be visually challenging^45–47^, and these conditions are associated with different outcomes.

##### Treatment response prediction

Radiomics with machine learning have been used to predict the response and outcomes of disease to treatment. Some examples of these include predicting the response of nasopharyngeal carcinoma to intensity-modulated radiation therapy^48^, the response of non-small cell lung cancer to neoadjuvant chemotherapy^49^, as well as the response to neoadjuvant treatment of rectal^50–52^, oesophageal^53,54^ and breast cancers^55,56^. Although highly promising, radiomics has not yielded widely generalizable results, thus limiting its current role and implementation in clinical practice.

##### Radiology-Pathology correlation

Matching radiology data to pathology report information is important for education, quality improvement, and patient care. Using natural language processing techniques, it is possible to mine text-based radiology^57^ and pathology^58^ report for key findings to cohort-specific populations for further investigative scrutiny. A system for natural language processing has been shown to classify free-text pathology reports (at an organ-level) to support a radiology follow-up tracking engine^59^, which can be used to alert radiologists to potential misses at study follow-ups. There also are opportunities to integrate anatomical pathology images with corresponding radiological images^60,61^.

##### Disease segmentation

The outlining of disease, or segmentation, is fundamental to many AI/ML and radiomics studies, and is necessary to derive quantitative tumour measurements including tumour diameters, as well as generating tumour contours for radiotherapy planning^62–64^. Registration of segmentations across time-series can also inform clinicians on how tumours are changing with treatment. Manual tracing of lesion borders can lead to high inter-reader variability^65^, which may be reduced with automatic disease segmentation using AI models. Although deep neural networks are powerful enough to segment lesions, it is recommended that the final AI segmentation result should be verified by an experienced radiologist.

**Fig. 4:**
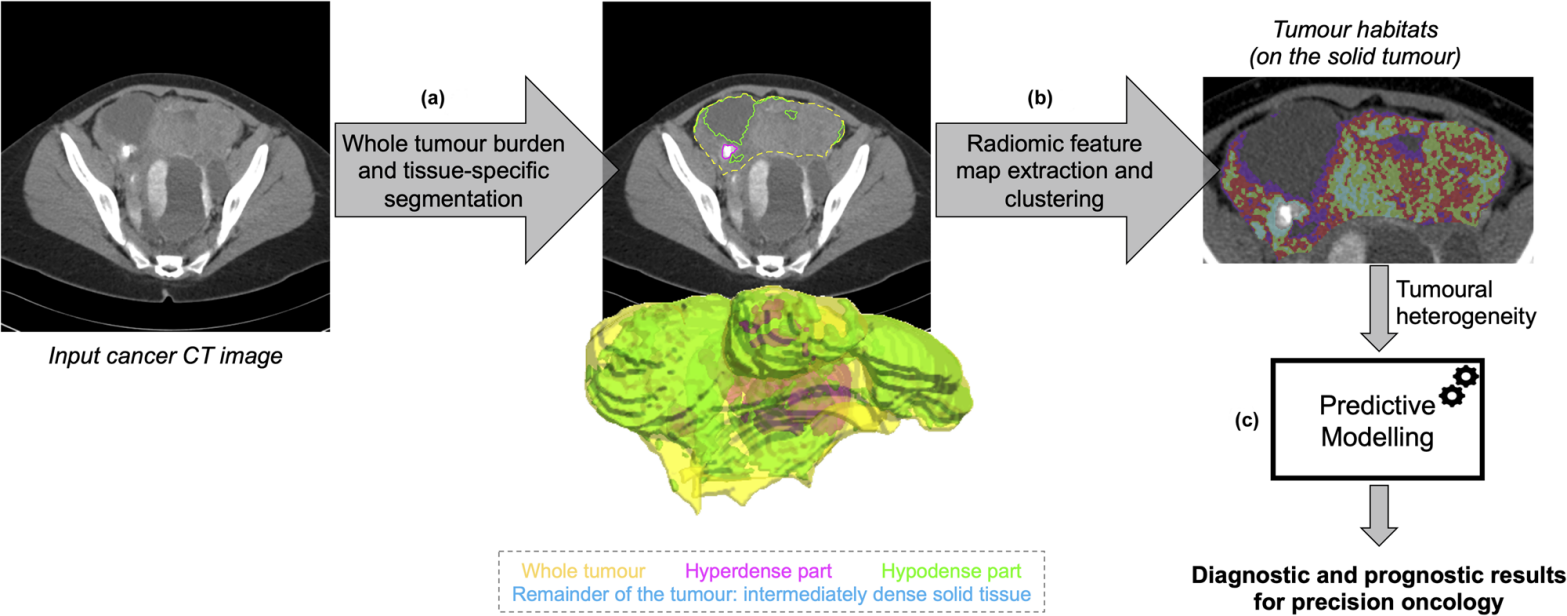
Machine Learning (ML) in a radiomics pipeline for evaluating tumour habitats. **a** Whole tumour segmentation and identification of physiologically different regions by means of tissue-specific sub-segmentation on computed tomograhy (CT) imaging (e.g. using 3D volume rendering of tissue components with colour codes shown below). This is followed by **b** voxel-based radiomic feature map extraction and unsupervised clustering for tumour habitats considering the most clinically relevant region. Next, **c** quantitative measurements and inferred tumoural heterogeneity metrics are processed by ML predictive models to yield diagnostic and prognostic results. In this example, we have used CT images from a patient with metastatic ovarian cancer with a representative omental lesion.

Segmentation algorithms are relatively well developed for certain image and disease types, probably due to the power of deep learning methods which have shown to be very efficient when sufficient data are available. A segmentation problem is a classification problem at the voxel level (a voxel being the smallest unit that makes up the image, determined by the image section thickness and the spatial resolution at which the image is acquired), and given the fact that lesions or whole organs are comprised of hundreds if not thousands of voxels, the density of the data is much higher compared with the classification problem usually considered at a per-patient level (e.g. radiomics). From the segmentation of the disease, radiomic features can be computed from the entire tumour, but a more sophisticated approach is to extract radiomic features from physiologically distinct regions (e.g. based on blood flow, cell density, necrosis) within tumours inferred by their imaging characteristics known as habitats^66,67^ (Fig. 4).

##### Organs-at-risk segmentation

The principle of radiotherapy is to inflict maximum damage to tumours while sparing normal tissues. However, normal tissues and organs often lie in close proximity to tumours, such that they are considered as organs-at-risk to the potentially detrimental scattering effects of radiotherapy. Organs-at-risk segmentation is necessary in radiotherapy to monitor and minimize radiotherapy damage to adjacent normal tissues. For example, when treating pelvic cancers^68,69^, organs-at-risk segmentation includes the outlining of the normal urinary bladder, bowel loops, rectum and both hip joints. ML has also been successfully applied in organs-at-risk segmentation for radiotherapy planning in head and neck cancers^70,71^, breast cancers^72^ and non-small cell lung cancer^73,74^.

##### Imaging optimization

One of the growing applications for AI and ML in imaging, not limited to cancer imaging, is their use for imaging optimization. For example, in MRI, the examination time of an oncological body examination can take 30–60 min. AI and ML techniques are increasingly applied to accelerate image acquisition and/or image reconstructions (i.e. making the examination faster)^75^; as well as to improve image quality (e.g. creating so-called super-resolution MRI images)^76^. The ability to shorten MRI examination time without sacrificing image quality can improve patient throughput to address bottlenecks in MRI capacity across health systems.

##### Others

Natural language processing is also being investigated as a tool to generate automated reports, and as a means of reducing repetitive tasks by radiologists^77^. For the clinicians receiving the radiology report, natural language processing can also potentially be used a communication tool to alert clinicians to actionable reports, so that critical findings can be highlighted to referrers in a timely fashion^78^.

The current relative success of AI and ML in the different use cases discussed above is dependent on the complexity of the undertaking, data quality and availability, the sophistication of the mathematical models and the subsequent real-world testing of the algorithms. Many of these use cases are active areas of research and development. However, algorithms that are being developed and tested may fail to translate into meaningful clinical tools. It is therefore important to understand the challenges and barriers that need to be addressed to enable the implementation of AI and ML in cancer imaging.

### Challenges for implementation of AI/ML in cancer imaging

While there are significant opportunities for the development of AI and ML in cancer imaging, there are also challenges to address. Below, we discuss some of the important clinical, professional, and technical challenges that will be encountered in the translation of useful mathematical algorithms into wider clinical practice for patient benefit.

#### Clinical challenges

One of most important considerations for the development of an AI or ML tool is that it should address a vital clinical challenge or question. As such, developers should have full appreciation of the clinical context and the implementation environment in which the AI tool is anticipated to operate. This will often require involving clinicians in the development of the tool.

The clinical domain is characterized by data inflow from different sources. The amount of biomedical data generated in the clinic is increasing due to advances in multi-modal imaging (i.e. imaging using a variety of techniques), high-throughput technologies for multi-omics (e.g. genomics, proteomics and molecular pathology), as well as an increasing amount of data stored within electronic health records. Hence, multidisciplinary engagement is critical to success. This complex and diverse information can potentially be integrated using AI and ML to support personalised medicine^79^. However, such large-scale datasets pose new challenges for data-driven and model-based computational methods to yield meaningful results.

AI has the potential to revolutionise cancer image analysis by applying sophisticated ML and computational intelligence. Cutting-edge AI methods can enable the shift from organisation-centric (based on organisational pathways) to patient-centric organization of healthcare, which may improve clinical outcomes and also potentially reduce healthcare costs^80^ by uncovering better individualized solutions. In addition, computerised oncological image analysis is encouraging the transition from largely qualitative image interpretation to quantitative assessment through automated methods aimed at earlier detection and enhanced lesion characterisation^81^, and the provision of better decision support tools. Within such a paradigm, there are important challenges that require better AI and ML solutions to solve. These include the need for reproducible and reliable tumour segmentation; accurate computer-assisted diagnosis; and clinically useful prognostic and predictive biomarkers with good performance. A particular challenge will be the quantification and monitoring of intra-/inter-tumoural heterogeneity throughout the course of the disease^82,83^. This will require access to high quality, longitudinal imaging datasets.

**Fig. 5:**
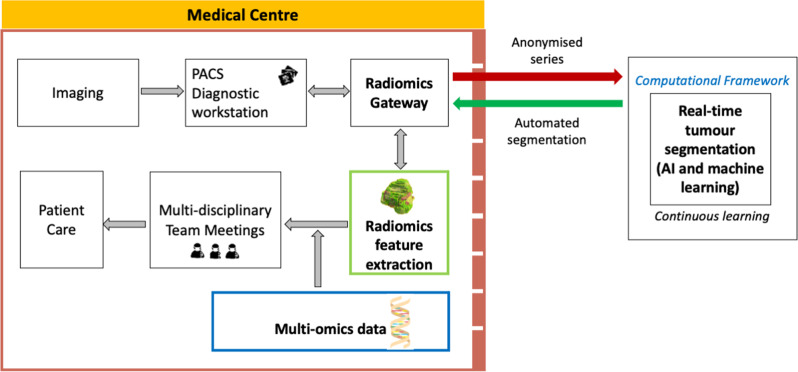
Potential future real-time tracking of whole tumour volume, spatial and temporal phenotypic heterogeneity with multi-omics data integration for precision oncology. This schema would allow the processing of multi-institutional data, where each medical centre acquires and stores (in local PACS) its own medical imaging data. To execute quantitative analyses, a radiomics gateway is used to communicate outside the institution by requesting an automated, real-time tumour segmentation from a trusted and specialised AI/ML centre, which allows for continuous learning. The medical images leaving the hospital are anonymised to deal with cyber-security and privacy issues. The segmentation results are used for radiomic feature extraction and analysis, acting as virtual biopsies. The quantitative imaging results are integrated with other biomedical data streams to determine associations with clinical and multi-omics information. Such an approach may develop reliable diagnostic and prognostic tools for multidisciplinary team meetings to improve cancer care in clinical practice; and the evolution of precision oncology. PACS Picture Archiving and Communication System, ML Machine Learning.

One area where AI/ML could be particularly transformative is precision oncology, or the selection of a patient’s therapy based on their tumour’s molecular profile. Precision oncology is likely to benefit from integrated diagnostics^84,85^ (e.g. radiogenomics, which combines radiomics and genomics analyses) to provide robust computational tools for investigating cancer biology, as well as for predicting treatment response (Fig. 5). The solution includes large-scale structured data collection (from multiple institutions) that deals with cyber-security and privacy issues and supports continuous learning. At present, the main challenge is bridging the gap between emerging AI tools and clinical practice, by first performing well-validated clinical research studies of such applications. This is vital for the translation and deployment of AI approaches in precision oncology^86^ and, if used correctly, AI has the potential to decrease the cost of precision oncological treatments through more accurate patient selection strategies.

### Professional challenges

Beyond the clinical challenges, there are professional challenges that are likely to shape the development and deployment of ML in cancer imaging. Stimuli promoting the development of ML include the relentless rise in the demand for imaging which, when coupled with acute and chronic workforce shortages, can lead to radiologist stress and burnout. Departments need to consider updating or redesigning their IT infrastructure and workflow to be ready for the testing and adoption of AI and ML technologies as these become available. Another challenge is how the radiological workforce perceives the potential utility of AI and ML in the clinic, including the threats and opportunities associated with the use of such technologies.

In preparation for an AI and ML in Cancer Imaging meeting organized by the Champalimaud Foundation (Lisbon) and the International Cancer Imaging Society in 2019, an online survey of 569 radiologists from 35 countries was conducted. The majority (>60%) perceived the benefits of AI to outweigh potential risks (Supplementary Note). Most respondents agreed with the positive impacts of AI in radiology, including (1) alerting radiologists to abnormal findings; (2) increasing work efficiency; (3) making diagnostic suggestions when the radiologist is unsure; (4) accepting that the radiologist should be responsible when an error is made; and (5) changing the service model by increasing direct communications with patients. The respondents felt confident that AI and ML techniques are unlikely to replace the job of a radiologist. The majority (>70%) felt that it was important to prepare for the arrival of AI by (1) investing in education; (2) testing new tools; (3) supporting the curation of images and image annotation data at scale; and (4) working with commercial vendors to develop specific AI tools that improve workflow.

The survey also identified areas of priority and need for AI tool development including the need for (1) tools that automatically track tumours across multiple time points to assess their response to treatment; (2) tools that improve automatic or semiautomatic tumour segmentation; (3) tools that support proforma reporting allowing annotation of image data to be captured prospectively; (4) tools that help confident identification of normal studies so that radiologists can focus on dealing with abnormal examinations; and (5) tools that help to identify tumours throughout the body.

In addition, imaging departments need to plan for their workforce needs to deliver future AI empowered practice. Radiographers and technicians will require better understanding of AI, including their deployment in workflow management and image acquisition. Critically, an informatics team is needed to create the platform on which AI tools can be developed or tested in-line; a space for interacting with and annotating imaging data; and well-curated imaging and data repositories.

### Technical challenges

Many state-of-the-art AI methods based on deep learning are achieving outstanding performance^87^. Reasons for their success include the strong ability of deep ML models to learn independently and the availability of large-scale labelled datasets that include precise annotations. Unfortunately, in biomedical research, collecting such accurate annotations is an expensive and potentially time-consuming process due to the need for domain experts’ knowledge^88^. Therefore, ML models that can work on rough annotations and weak supervision (e.g. bounding boxes that encompass an area of interest rather than precise outlining, or image-level labels rather than specific image-feature labels) have been attracting much attention^89^. The generation of large mineable imaging datasets might overcome data paucity and heterogeneity issues. However, along with the availability of samples, data quality and diversity should be considered by collecting and preparing harmonized datasets. The ability to generalize across multi-institutional studies may be improved by exploiting transfer learning and domain adaptation techniques.

Designing and identifying reliable AI imaging studies is a challenge. Studies have been published with as few as 10 patients, making the results of such AI models highly questionable due to potential overfitting effects, which will negatively impact upon the generalizability of the findings. In radiomics, there is a rule of thumb when dealing with binominal classification tasks where 10–15 patients should be recruited for each feature that is part of the final radiomics signature^90^. Performance estimation should be based on the so-called test set: that is, a dataset comprised of examples that were completely excluded from the model’s training and tuning processes. To evaluate the model’s generalizability, apart from internal validation, external validation should be performed to test the model’s performance in one or more datasets acquired using different imaging equipment or in different geographical patient populations. Ideally, models should be validated in an external patient cohort that is 25–40% of the size of the training sample.

Integrative models fusing information from other omics data such as genomics or proteomics, as well as clinical, environmental and social data, are gaining attention, especially in the setting of more complex clinical problems such as disease risk assessment and prognosis. Data sparsity and non-standardized therapeutic approaches between institutions are ongoing challenges when it comes to developing integrative ML models, but there is recognition of the need for better standardization (including data acquisition) that will facilitate these use cases of AI^91^.

The use of images and integrating these with clinical and molecular data can be a source of real-world data to be used for evidence-generating studies. Retrospective data from imaging biobanks and repositories provide excellent opportunities to test AI tools and validate their performance. Harmonization techniques like ComBat^92^ can be considered to bring the imaging features into a standardized space, especially in multicentre studies when the amount of variability, if not reduced, can harm a model’s performance and generalizability. Radiologists have an excellent opportunity to lead the field by promoting observational in silico studies, taking care to oversee all relevant aspects from data harvesting to analyses to improve the reproducibility of results. The main aspects to be considered are as shown in Box 1^93^.

For the specific application in radiomics, there are also many challenges to radiomic computation and the use of radiomic features for prognostication, assessment of response to therapy, and diagnosis of molecular phenotype, including the sensitivity of radiomic feature values to image acquisition and reconstruction techniques^94–98^ and to variations in segmentations among different users and software^99,100^. To address these points, improvements in algorithms, and community agreement on use of open-source software, phantoms and standardized approaches^101^ are required for radiomics to reach its full potential.

One of the reasons for the lack of translation of AI models to clinical application is that the focus has been on increasing model performance by AI enthusiasts, possibly at the expense of explainability. A typical example is the black-box approach of deep neural networks that produces outstanding performance, but may present difficulty in establishing its trustworthiness, therefore impeding its clinical adoption. A lack of multidisciplinary engagement may also impede the prioritization of AI solutions of significant clinical value. The clinical community may be skeptical about embracing AI technology into clinical routine, as long as the AI models are non-transparent in the way they reach a specific decision.

In recent years, the AI community has started to recognise this limitation and has moved towards the development of explainable AI. The explainability of AI models touches upon a sensitive issue concerning patient safety, especially in clinical decision-support systems^102^. Since the vast majority of AI models are trained with retrospective, observational data, patient selection bias in machine learning models can lead to poor performance and erroneous predictions in prospective unknown cases. Therefore, domain experts should always verify the predictions and the reasoning behind the predictions made by the AI models. The latter can only be achieved when the models by design offer a degree of transparency. Involving the domain expert in model development is likely to make AI models more robust and reproducible and help gain the trust of end-users. Evaluating the overall performance of the AI solution beyond accuracy is also mandatory in the clinical pathway setting. This would include testing the real-world implementation of such models to ascertain their use and usability, trustworthiness, as well as cost and cost-effectiveness.

Box 1 Important considerations from data curation to analyses to improve the robustness and generalizability of AI and ML in cancer imaging

- Participant recruitment criteria
  Consistency in the inclusion of the study population based on the presenting symptoms, results from previous tests, defining the appropriate index tests or by the selected reference standard
- Participant sampling
  To avoid or control bias in participant sampling, considerations could include the use of consecutive series of participants, use of well-defined selected data silos, clear and well-defined selection criteria; as well as adjusting for possible confounding variables
- Data collection
  What data to collect and how this is performed should be planned before participant recruitment and sampling. Where appropriate, target trial emulation may be undertaken, which is the application of design principles from randomized trials to the analysis of observational data, which may improve the quality of the observations.
- Reference standard
  The rationale and description of the reference standard should be clear
- Technical specifications of materials and methods
  Aspects of technical specifications should be well defined. These include how and when images and measurements were taken; the definition of units; cut-off thresholds; defined results categories (of both the index tests and the reference standard); description of the number, training, and expertise of persons executing and reading (original or new reporting); index tests and the reference standard; and blindness aspects (if the readers of the index tests and the reference standard were masked to other test results)

### Lessons learnt from the past: computer-aided diagnosis (CAD) for breast cancer

Even though AI and ML are hugely promising technologies in imaging, it is worth noting lessons from the previous effort to apply computational approaches in cancer imaging, using computer-aided diagnosis of breast cancer as an example. Development of algorithms for automated detection of calcifications and masses on mammograms started in earnest in the mid-1980s, and in 1998 the first commercial CAD system for mammography, initially based on digitized film, received FDA approval^103^. Transition to digital mammography facilitated the implementation of CAD in clinical practice. The introduction of Medicare reimbursement coverage for the use of CAD in the United States, and promising preliminary results from clinical trials^104,105^, led to a rapid uptake of CAD in the US with ~74% of mammography interpretations utilizing CAD by 2010^106^. However, even though stand-alone sensitivity of commercial CAD systems in enriched reader studies is consistently superior to that of radiologists^106^, large retrospective registry-based studies failed to show a significant improvement in the diagnostic accuracy of screening mammography after implementation of CAD^107,108^. This disappointing result is likely to be explained by the relatively high number of false-positive prompts generated by current commercial CAD systems, which average between 1 and 2 false prompts per case. In the low-prevalence screening setting, this false-positive prompt rate translates into a positive predictive value of a CAD prompt of <1%. As radiologists will have to ignore more than 99% of the CAD prompts to find the one prompt actually pointing to a cancer, there will be a tendency to ignore the computer-generated prompts altogether. There is hope that newer deep learning algorithms will overcome some of the limitations of traditional feature-based CAD systems. Unsupervised training on much larger datasets with up to a million mammographic images has the potential to overcome the shortcomings of human observers, as deep learning algorithms no longer have to imitate the way the radiologist reads a mammogram^109^. However, increased automation of the detection task will come with added responsibilities for the algorithms^110^, which may need to show an improvement in patient outcome beyond diagnostic performance.

Hence, the key lessons from previous CAD implementation in breast cancer suggest that the next generation AI tool to for cancer detection will need to have high diagnostic accuracy, in particular, high positive predictive value that will result in fewer false positives in the low disease prevalence setting. There is also the need for real-world testing of these tools beyond diagnostic performance to establish the health-related and wider benefits associated with their deployment.

### Technical, infrastructure and professional developments required for the adoption of AI/ML in cancer imaging

#### Imaging repositories and archives

Supervised learning approaches require large quantities of labelled data for training and validation^103^. There is a plethora of data sources that one could exploit for AI modelling in cancer imaging. These include imaging biobanks, which are virtual repositories of medical images; imaging biomarkers identified as endpoint surrogates; and population studies^111^. Imaging biobanks allow the in silico evaluation and validation of new biomarkers by establishing disease development probabilities, early disease diagnosis and phenotyping, disease grading and staging, targeting therapies and evaluation of disease response to treatment and prediction of adverse events.

Open access data repositories are one approach to capturing and disseminating sufficient high quality, well curated data. There are not many open access cancer image repositories. Data sharing is not a universally accepted concept^112^. Furthermore, patient privacy, data privacy, informed consent laws, regulations and the growing interest in the potential commercial value of patient data, differ by country and can pose barriers to data sharing^113^. Institutions and researchers consider data to be intellectual property, and limit or prohibit access to valuable data sets. Regulatory agencies (e.g. the FDA) argue for sequestration of data used to validate algorithms approved for commercial use^114^.

**Fig. 6:**
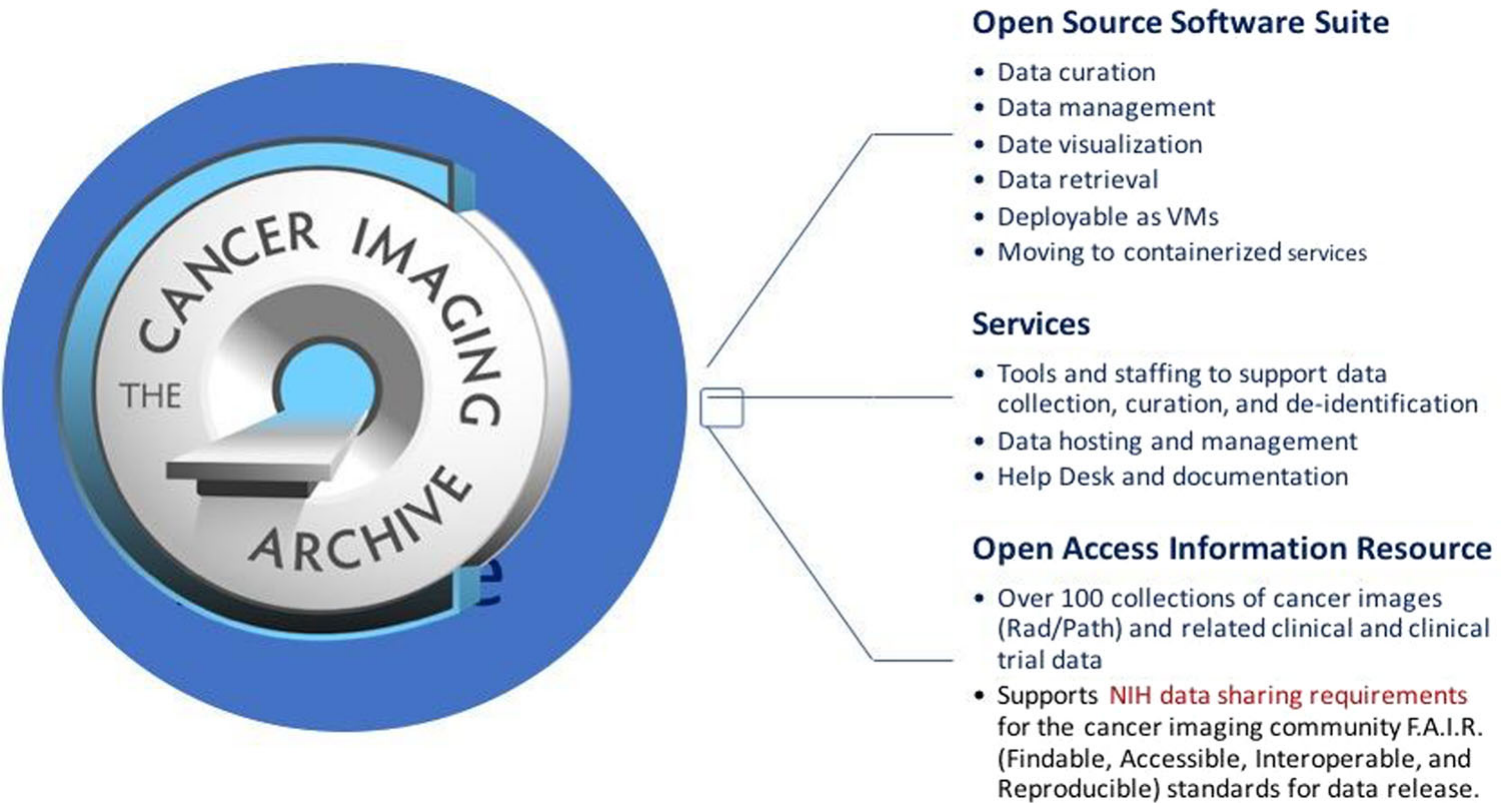
The Cancer Imaging Archive (TCIA) is a system of systems constructed from open-source software. TCIA is also a set of services designed to collect and curate high quality cancer image data and related clinical data and make it publicly available. (VMs = virtual machines).

The US National Cancer Institute funded the creation and continued operation of the largest open access cancer image repository, The Cancer Imaging Archive (TCIA) (Fig. 6)^115,116^. TCIA is designed to foster increased public availability of high-quality cancer imaging data sets for research. Data are accessible due to strict adherence to F.A.I.R. (Findable, Accessible, Interoperable, and Reproducible) standards for data release^117,118^. Other research-funded initiatives to create data warehouses are also being developed across the European Union and elsewhere.

Although size of a dataset matters, data quality and data variability are of equal importance. Data should be of sufficient quality and be acquired with uniform parameters. Clinical trials generate data with a higher level of quality control and consistency of data acquisition protocols. TCIA focuses on collecting, curating and publishing data from completed clinical trials. Curation in this context includes assurance of consistent metadata, anatomy coverage, and data formats which strictly comply with international data standards, as well as the anonymization of any patient identifiable data.

For an ML algorithm to be clinically useful it must be trained on data that appropriately represent the variance in the human population, the presentation of disease and data collection systems^119,120^. Labelled data are created manually by human experts, resulting in high cost and limited volume of high-quality training (and testing) datasets. Perhaps the most time-consuming process within a ML project is annotating the data and presenting them in a format compatible with further analysis and modelling processes. Image annotation is often a bottleneck for AI and ML, and crowd sourcing for such activity is being trialled as a way of improving efficiency. Depending on the task, the annotations may be provided at the patient level (overall survival, disease-free survival), at the image level (benign, malignant) or at the voxel level (lesion, non-lesion). Typically lesion detection algorithms need to be provided with annotations of a bounding box type usually encasing the lesion, while for training automatic segmentation models, radiologists need to outline lesions manually in multiple image slices^121^.

As sizeable imaging data from different sites and scanners become consolidated within repositories, it will be necessary to consider steps that will account for data diversity or heterogeneity. A possible solution might be to use deep learning approaches to learn from such data lacking homogeneity, which may result in outputs with lower variability and higher reproducibility. Retrospective observational studies with real-world data and quality assurance checklists^93,122^ will allow reproducible causality^123^ inferences from virtual patient cohorts to address clinical and policy-relevant questions. Particularly where the disease under study is relatively rare resulting in small datasets, it would be appropriate to use a cross-validation approach to develop and test the AI models.

#### Open-source software and open collaborations

The use of open-source software (OSS) strategy could help to alleviate some of the concerns regarding transparency and explainabiltiy when using AI in cancer imaging. OSS is software code made available under a legal licence in which the copyright holder provides (depending upon the specific terms) various rights to the licensees to study, change, improve and re-distribute the code without any fees. Today, there are many different types of OSS licences [https://opensource.org/approval] depending on the preference of the copyright holder. These licences range in the United States from what is commonly known as permissive licences, such as Apache-2.0, to strongly protective licenses, such as general public licence (GPL). OSS is available in its non-commercial form, however it can be made into commercial products with additional services such as warranty, training, documentation and maintenance under various commercial contracts.

A successful open-source ecosystem has three interacting components: (i) OSS itself, (ii) governance, and (iii) community of collaborators. Currently there are more than 50 open-source ML packages using different OSS licences, operating platforms, and programming languages. Some of the more popular packages include TensorFlow, Keras, PyTorch, Caffe2 and many others. They all have varying strengths and weaknesses depending on users’ needs.

These OSS packages are developed and sponsored by corporations and some individuals for their own use cases and applications, often not for medical imaging, but the packages are good initial platforms from which medical imaging research can be pursued. However, they will need to be optimized for higher performance for medical applications. For example, the pattern recognition in consumer applications usually depends on graphic features and image orientation. However, medical image patterns are usually orientation-independent, and diseases in medical images are subtle in nature and present themselves in minor grey value differences rather than graphical features. For these reasons, algorithms available on OSS packages will need to be re-trained and tuned using medical imaging data to optimise their performances. In summary, OSS represents a practical route by which the AI community can work together to collaborate and develop new AI tools, which can be more widely tested, and at the same time address some of the transparency and privacy concerns.

#### Healthcare and regulatory systems

There are significant perceived values of using AI solutions in healthcare^124^ at every stage of the clinical workflow. In radiology, this means improvements to the patient diagnostic pathway, from the appropriateness of imaging requests^125^ to how actionable findings in radiological reports are followed up^126^. The full potential of these improvements are not yet realised as there remain significant barriers to implementation.

From 2021, the new EU Medical Device Regulations has been enforced, mandating deeper scrutiny of software as a medical device (SaMD). Certification is given in accordance with how the software is used and applied within the clinical workflow. The majority of AI software in imaging are being certified as a decision-support tool, that is to say it should not be used on its own in for clinical or patient management. It is also worth considering whether the software is intended to be use by radiologists at primary reporting, or only after the initial primary report is issued as a second read. In the current commercial landscape, there are multitudes of software tools that are cleared by regulators but have not been adopted into healthcare systems.

AI products may continue to evolve after initial release through continuous training. Many products have found their way into the marketplace without being independently tested, despite obtaining CE labelling or FDA clearance. As such, a new FDA framework has been proposed to ensure the safety and effectiveness of AI tools^127^. The FDA has introduced a predetermined change control plan in premarket submissions. This plan includes anticipated modification (SaMD pre-specifications) and the associated methodology used to implement these controlled changes (algorithm change protocol). The FDA expects a commitment from manufacturers on transparency and real-world performance monitoring, as well as updates on changes implemented as part of the approved pre-specifications and the algorithm change protocol.

Once the product or software has been validated as a certified medical device, a Data Protection Impact Assessment process must be initiated, usually at the local level, to safeguard data privacy—in Europe, this means compliance with the General Data Protection Regulations (GDPR). At the same time, a Solution Architecture Review should also be undertaken to carefully examine the possible IT architecture for implementation. Local rules must also be adhered to with regards to patient data use and storage, since each country can vary in the interpretation of the GDPR. Privacy concerns and the need for a rational and coherent digital infrastructure has been referred to as ‘the inconvenient truth’ in medical AI^128^.

The process of software integration with existing hospital IT infrastructure is influenced by the experience of the AI company and its product design, the diversity and size of the healthcare system, as well as knowing how and what data are being transferred to and from the healthcare provider to the software processor and vice versa. Failure of software integration is a known barrier for adoption. Well-established companies with a sound product could be integrated in days, but the timeline usually gets longer in hospitals running an array of different radiology informatic systems (e.g., Picture Archiving and Communication Systems [PACS] and Vendor Neutral Archives [VNA], which communicate with the Hospital and Radiology Information Systems [HIS & RIS],) as well as dealing with a complex range of data inputs (e.g. non-standardised naming of imaging sequences from different scanners).

To facilitate AI workflows, similar imaging procedures should be standardised to the same acquisition protocol (regardless of scanner model and vendor), and all radiological reports could be structured in a similar way using common lexicon to facilitate data mining (e.g. RadReports.org with suggested structured reporting templates endorsed by the American College of Radiology). Without satisfying such conditions, software integration may need to be organised on a per-modality basis, which may require complex data mapping within the same hospital system. Hence, depending on how mature the software algorithm is, program bugs may reveal themselves as a consequence of such data input heterogeneity.

Introducing the use of a new AI tool within a healthcare system may proceed with initial caution by working with the supplier to undertake a mutually agreed trial period. Such a “try to buy” approach would allow users to assess the use and usability of the AI tool, integration with the workflow, as well as its trustworthiness. This is because physicians may distrust the tool unless it is proven to be highly accurate. One solution is to build a radiologist feedback tool onto the PACS interface. This would allow the radiologist to score the performance of any given AI algorithm—for example, using check boxes with legends such as ‘agree/AI overestimation/AI underestimation/both over and underestimation’. This would allow users to raise perceived discrepancies that can then be further assessed. Caution should also be given to tools that are developed by vendors that may lock-in users to specific algorithms, especially if they fail to meet local demands. The community of professionals who interact with the software tool also needs to be educated about its usage. It may be feasible for an AI developer to train a small group but this becomes challenging when confronted by many potential users in a large hospital system.

It is possible to process patient data using certified medical devices in routine clinical practice without additional consent. However, if vendors are seeking feedback to improve their software algorithm, then specific data consent is required and should be obtained prospectively from patients. Post-hoc sharing of such data may be denied, which means that processes must be put in place to identify patients who have provided consent and to rescind it where appropriate.

Even when the barriers to AI implementation are overcome, it may still be unclear: who pays for the AI? Whilst the development and testing AI tools can be funded by research grants or commercial partnership with companies, as yet, no healthcare systems or private health insurers have reimbursed AI usage. In the landscape of decreasing tariffs for radiological procedures, it is a challenge to find specific funding to support the introduction of AI, which can be costly to deploy across healthcare systems. Even though AI holds substantial promise to improve work efficiency, there are yet no published real-world evidence to date. The development of specific patient-centric services using AI may provide an opportunity to introduce tariff models for its use. One example is the UK pilot of a bone health service, which pays for identifying patients at risk of developing osteoporotic spinal fracture. Instead of payment for a specific AI product, the business case was constructed on the basis of the whole service, which aimed to identify patients at risk of osteoporotic fracture, thus enabling early intervention and potentially reducing subsequent healthcare costs by decreasing the number of fractures. This is an example of the coming together of value-based healthcare and AI.

In less coherent healthcare models where imaging services are component care providers (i.e. providers of specific services), it would be important to accrue local metrics to help justify AI adoption. Examples of these include metrics showing improvement in the accuracy of reporting by reducing the rate of patient recall in women undergoing mammography^109^; increasing the reporting speed and finally increases in revenues. By testing novel AI solutions in a variety of healthcare markets and trying different combinations of payor models, it may eventually be possible for AI software tools to be widely adopted into healthcare systems (Box 2).

#### Future radiological workforce

Appropriate training is required to allow users to judge whether an AI tool is fit for purpose before adoption into clinical practice, which would require radiologists to understand the principles of AI and how AI algorithms should be properly validated.

There are pressures that are encouraging the premature introduction of AI tools into clinical workflows. Firstly, there is a workforce crisis with a shortage of radiologists in many countries. In the UK, about 10% of radiologist vacancies are unfilled^129^. Secondly, there is a marked increase in global imaging demand and workload. In the UK, the CT and MRI workload has been rising by ~10% each year^129^. Thirdly, there is a relentless drive to improve workflow efficiency, by improving image procedure turnaround time without compromising diagnostic accuracy. Finally, AI is seen as a tool to support repetitive tasks (e.g. sequential tumour size measurement, or cancer screening), that are time-consuming and relatively uninteresting for radiologists to undertake.

Empowering radiologists to judge the performance of AI algorithms would require changes in medical school and radiology curricula to include an understanding of the terms and main methodology of AI/ML; the requisite development, training, testing and validation of algorithms; basic statistics relevant to AI/ML; and the challenges of data requirements. Such empowerment will also necessitate educating radiologists in how they can meaningfully and rigorously test the performance of AI algorithms within their own clinical practice.

The future of AI and ML applications in radiology will be reliant upon the education of stakeholders including medical students, trainee radiologists, qualified radiologists, other doctors, radiographers, computer scientists, data scientists and data engineers collaboratively to solve clinically relevant problems. This multidisciplinary dialogue is necessary and critical to the development of clinically relevant and technically accomplished AI tools to address the unmet needs in oncology. There is a clear need for more multidisciplinary AI meetings and conferences to encourage interactions between all stakeholders, both at the local level, as well as at the national and international level.

Box 2 Important factors for the selection of AI into a health system *Criteria and benchmarks*

- CE labelling
- FDA clearance
- UKCA marking

*Incentives and motivations*

- Targeting a common disease
- Potential for the AI algorithm to be developed into products that generate revenue
- Attracting better or new payors
- Formulation of fair value proposition between stakeholders or partners
- Latitude to create/share own business model
- AI tool Infrastructure fits with existing informatic systems
- The AI tool can be assimilated into the clinical workflow

## Conclusions

Cancer imaging is seeing rapid developments in AI, and in particular ML, with a broad range of clinical applications that are welcomed by the majority of radiologists. The development of new ML tools is often constrained by available imaging data; however, there is the potential for building and using real-world well-curated imaging data in biobanks and open access repositories to overcome such limitations. Adopting open-source tools for algorithm development, where possible, may lead to better transparency and collaboration across centres. However, even though exceptional diagnostic performance can be gained by the application of these AI software algorithms, it is still not clear how many of these will have a long-term meaningful impact on patient outcomes or will be cost-effective. An improved regulatory framework for the approval of AI-based tools for clinical deployment is evolving. There is a need for systematic evaluation of these software, which often undergo only limited testing prior to release. It is also important to empower all stakeholders, especially radiologists, with sufficient understanding of this growing field to enable them critically to appraise these technologies for adoption into their own practice. Creating opportunities for interdisciplinary engagement will also facilitate the development of useful clinical tools that aim to enhance patient care and outcomes.

## Supplementary information

Supplementary Information
