# Supplementary material for: Artificial intelligence and machine learning in cancer imaging
Source: Commun Med (Lond). 2022 Oct 27;2:133. doi: 10.1038/s43856-022-00199-0 (PMC9613681; doi:10.1038/s43856-022-00199-0)
Supplement: Supplementary file 1 — Supplementary Information [file 43856_2022_199_MOESM1_ESM.pdf]

Supplementary Note

The survey was undertaken by the International Cancer Imaging Society. The Society is registered as a Charity in the United Kingdom (UK). The survey was conducted in compliance with the principles of good practice in the management and conduct of health and social care research in the UK.

The aims of the study were to understand the prevailing attitudes of radiologists and allied healthcare professionals towards artificial intelligence and machine learning as related to imaging; the perceived challenges facing imaging practices which may be facilitated by artificial intelligence and machine learning; to understand which priorities should be targeted and how imaging departments could prepare for these changes.

Informed consent:
Written informed consent was obtained from all subjects/participants, by agreeing to participate in the survey.

Ethical approval
Institutional Review Board approval was not required because the study was a voluntary survey among radiology professionals not related to any specific health information and all data was handled anonymously. No vulnerable individuals participated in the survey. Participants were informed that the data would be collated anonymously for analysis and dissemination.

The survey was undertaken using SurveyMonkey. An open invitation was sent via Twitter and email outreach through personal contacts of the co-authors and through world-wide national radiological societies to participate. The survey was opened for a period from November 2018 to end of April 2019, a period of 6 months.

The full survey is a subject of a separate manuscript and hence only the items relevant to the information presented in this manuscript are presented here.

Reponses to survey
The survey elicited 569 responses from radiologists from 35 countries. Males accounted for 62% of respondents, 38% were females. The mean age was 45 years.

Survey questions and findings
Respondents were asked: “Which of the following is closest to your view about the balance of risks and benefits with AI/machine learning being used in imaging?” with choices as follows:
a. The benefits are much bigger than the risks
b. The benefits are slightly bigger than the risks
c. The benefits and risks are both equal
d. The risks are slightly bigger than the benefits
e. The risks are much bigger than the benefits

>60% of the respondents indicated choice (a) or (b).

Respondents were asked the extent to which they agreed or disagreed with a number of known perceptions/viewpoints that were positive or negative towards AI. For each statement, respondents indicated whether they strongly agree, agree, neither agree or disagree, disagree or strongly disagree.

The statements and the percentage of responses (in brackets) with which respondents strongly agree or agree are as below:

- Alerting radiologists to abnormal findings (94%)
- Increasing work efficiency (81%)
- Making diagnostic suggestions when the radiologist is unsure (68%)
- Accepting that the radiologist should be responsible when an error is made (58%)
- Changing the service model by increasing direct communications with patients (56%)

In addition, the majority (67%) disagree or strongly disagree that AI and machine learning could replace the job of a radiologist.

Respondents were asked the extent they agree or disagree (using a similar scale as above) with the steps needing to be undertaken to prepare for the arrival of AI in their own department. The majority (>70%) felt that it was important to prepare for the arrival of AI by (1) investing in education; (2) testing new tools; (3) supporting the curation of image and image annotation data at scale and (4) working with commercial vendors to develop specific AI tools that improve workflow.

Respondents were also asked the extent they agree or disagree with the areas needing to be developed and strong support (>70%) was expressed for: (1) tools that automatically track tumours across multiple time points to assess their response to treatment; (2) tools that improve automatic or semiautomatic tumour segmentation; (3) tools that support proforma reporting, allowing annotation of image data to be captured prospectively; (4) tools that help confident identification of normal studies so that radiologists can focus on dealing with abnormal examinations and (5) tools that help to identify tumours throughout the body.
